# Prescribing Blood Flow‐Restricted Cycling Using Rating of Perceived Exertion Balances the Physiological and Perceptual Demands in Young Healthy Adults

**DOI:** 10.1002/ejsc.70009

**Published:** 2025-07-16

**Authors:** Nathan D. W. Smith, Brendan R. Scott, Olivier Girard, Jeremiah J. Peiffer

**Affiliations:** ^1^ School of Health Sciences University of Notre Dame Perth Australia; ^2^ Centre for Healthy Ageing Murdoch University Perth Australia; ^3^ Physical Activity, Sport, and Exercise (PHASE) Research Group School of Allied Health (Exercise Science) Murdoch University Perth Australia; ^4^ School of Human Sciences (Exercise and Sport Science) The University of Western Australia Perth Australia

**Keywords:** aerobic exercise, discomfort, effort, moderate intensity, pain, vascular occlusion

## Abstract

To compare the physiological and perceptual responses during fixed‐power and perceptually regulated cycling, both without and with blood flow restriction (CON_PWR_, BFR_PWR_, CON_RPE_ and BFR_RPE_). Twelve recreationally active men cycled for 10 min at the power corresponding to the first ventilatory threshold or, for CON_RPE_ and BFR_RPE,_ the perceived exertion level reported during CON_PWR_. Blood flow restriction was set at 60% of estimated arterial occlusion pressure. Ventilatory measures and heart rate were averaged into 2‐min blocks. Perceived exertion, effort, muscular discomfort and cuff pain were recorded every 2 min (0–10 scale). Blood lactate was measured pre‐exercise, post‐exercise, and 2 min post‐exercise. The BFR_PWR_ trial elicited greater physiological and perceptual responses compared to all other conditions. Oxygen consumption during BFR_RPE_ was lower than CON_PWR_ (−19.2 ± 20.6%, *p* < 0.001) and CON_RPE_ (−6.7 ± 9.3%, *p* = 0.007). Heart rate during CON_PWR_ was greater than BFR_RPE_ (8.2 ± 9.8%, *p* < 0.001) and CON_RPE_ (9.4 ± 6.5%, *p* < 0.001). Blood lactate concentration was not different between CON_PWR_, CON_RPE_ and BFR_RPE_; yet was greater during fixed‐power compared to fixed‐RPE trials (31.5 ± 25.6%, *p* < 0.001). Muscular discomfort was not different between BFR_RPE_ and CON_PWR_ (2.4 ± 1.1 au), yet both were greater compared to CON_RPE_ (1.8 ± 1.5 au, *p* < 0.001). Cuff pain was greater during BFR_PWR_ (3.3 ± 1.7 au) compared to BFR_RPE_ (2.2 ± 1.1 au, *p* < 0.001). Prescribing aerobic BFR cycling at a fixed power output increases physiological strain, yet discomfort and pain are also heightened, which may limit its use in healthy adults. The fixed‐RPE method appears to balance the physiological and perceptual demands and thus could be a viable alternative if a fixed power output approach is intolerable.

1

**Summary**

- The selection of a fixed‐power or fixed‐RPE prescription method for aerobic BFR exercise depends on the desired physiological stress and likelihood of adherence.
- Fixed‐power cycling with BFR at the first ventilatory threshold was associated with the ‘heavy’ exercise domain in some participants, and thus caution is required when prescribing such exercise to some clinical populations.
- The fixed‐RPE approach likely reflects the overall physiological and perceptual demands, and as such is a convenient and suitable method of incorporating aerobic BFR exercise into a comprehensive training program.

## 2 Introduction

Physical inactivity is a major contributor to poor health (Lee et al. 2012), with a lack of time often cited as the main reason for not meeting the minimum guidelines for improving health and decreasing all‐cause mortality (Hoare et al. 2017; Burton and Turrell 2000; Garber et al. 2011). For individuals such as those with musculoskeletal injuries or older adults, physical limitations can make it more difficult to meet physical activity guidelines. Implementing strategies which enable these individuals to meet the recommended guidelines in a time‐efficient manner could significantly improve their health. Current literature indicates that the use of blood flow restriction (BFR) during aerobic exercise presents a viable modality to address this challenge (Silva et al. 2019; Cognetti et al. 2022). For instance, eight weeks of BFR cycling, consisting of three weekly 15‐min cycling sessions at 40% maximal oxygen uptake, has been shown to improve isometric quadriceps strength by 7.7% and maximal oxygen uptake by 6.4% in recreationally active males (Abe et al. 2010). Such improvements are not typically observed with training at such low absolute exercise intensities and volumes, nor are concurrent improvements in strength and aerobic fitness generally expected from a single exercise modality.

It is common practice to prescribe aerobic exercise at a fixed speed or pace, power output, or heart rate to improve aerobic fitness (Garber et al. 2011). However, BFR induces localised hypoxia that increases peripheral fatigue while decreasing venous return, which can reduce speed/power or elevate heart rate, respectively (Ozaki et al. 2010). These changes complicate the accurate prescription of aerobic BFR exercise intensity. The use of BFR at fixed workloads has been shown to shorten time to task failure (Sakamaki‐Sunaga et al. 2012), cause an inability to complete an exercise session (Kilgas et al. 2022) and result in the withdrawal from a training intervention (Kim et al. 2017), all of which reduce exercise adherence, which may limit fitness improvements. An alternative is a perceptually regulated approach using ratings of perceived exertion (RPE) instead of fixed metrics such as power output or heart rate. Outside of the BFR literature, prescribing high‐intensity interval training and moderate‐intensity continuous training using a perceptually regulated approach have improved maximal oxygen uptake in nonathletic cohorts (Soylu et al. 2021; Connolly et al. 2017). In trained individuals aerobic BFR exercise can be effectively prescribed using RPE (Smith et al. 2024). The same RPE reported during fixed‐power cycling without BFR was applied to prescribe BFR cycling to trained cyclists, which resulted in comparable heart rate (140 ± 14 vs. 139 ± 16 beats·min^−1^) and post‐exercise blood lactate (3.9 ± 1.8 vs. 3.9 ± 2.1 mmol·L^−1^) between the two conditions (Smith et al. 2024).

As RPE reflects both peripheral fatigue state (Amann et al. 2013) and physiological strain (G. Borg et al. 1987), an increased RPE at fixed workloads with BFR likely represents the added training stress rather than being solely a consequence of BFR itself. However, non‐athletes might find RPE‐based regulation challenging due to an unfamiliarity with the task (St Clair Gibson et al. 2006; Tucker 2009). This could be further exacerbated by the application of BFR due to the associated localised hypoxia, blood lactate accumulation and perceptual sensations of discomfort and pain that can influence whole‐body RPE (Kilgas et al. 2022; Nicolò et al. 2014; Jeffries et al. 2019). As such, non‐athletes may struggle to match the physiological demands of RPE‐based exercise with BFR to the same task without BFR. Nevertheless, RPE‐based regulation could help individuals modulate discomfort from BFR by reducing intensity, enabling completion of the entire exercise task. Determining how BFR influences RPE‐based regulation of aerobic exercise in healthy adults, therefore, warrants further investigation to inform exercise professionals of the advantages and disadvantages of this prescription method for BFR exercise in this population.

The purpose of this study was to compare the performance (power output), perceptual (RPE, effort, discomfort and pain) and physiological (heart rate, respiratory frequency and oxygen uptake [V̇O_2_]) responses of cycling with and without BFR during both fixed‐workload and perceptually regulated prescription models. It was hypothesised that applying BFR would result in greater increases in physiological and perceptual responses during a fixed‐power model compared to a perceptually regulated prescription model.

## 3 Methods

### 3.1 Experimental Approach to the Problem

Participants completed a preliminary visit and four experimental sessions, all separated by at least 24 h and conducted at the same time of day (± 2 h). Experimental sessions involved cycling for 10 min using two methods of prescribing exercise intensity, fixed‐power (PWR) and fixed‐RPE, each with and without BFR (BFR and CON): BFR_PWR_, CON_PWR_, BFR_RPE_ and CON_RPE_. Both BFR_PWR_ and CON_PWR_ were prescribed at the power output associated with the first ventilatory threshold. The RPE prescribed during BFR_RPE_ and CON_RPE_ was matched to the RPE reported during CON_PWR_. The experimental design therefore required CON_PWR_ to be performed first, followed by the remaining three experimental conditions, which were completed in a randomised, counterbalanced order. Participants were asked to refrain from alcohol, caffeine and strenuous exercise during the 24 h prior to each visit.

Twelve healthy recreationally active (McKay et al. 2022) males participated in this study (age: 28 ± 7 years; body mass: 81.6 ± 12.7 kg; stature: 177.0 ± 8.0 cm; weekly activity: 456 ± 261 min·week^−1^). Their regular exercise included running, team sports and resistance training. The sample size of 12 was calculated using G × Power (version 3.1.9.7) to detect an effect size of *ƒ* = 0.35 using a within‐subject repeated measures analysis of variance (*α* = 0.05, 1–*β* = 0.8). The study's purpose and requirements were explained to participants before obtaining written informed consent. Individuals were excluded if they indicated haematological, musculoskeletal or neuromuscular abnormalities or were taking medications likely to influence the main outcome measures. Ethical approval was obtained from the institutional ethics committee (ref: 2021/054). Only one sex was recruited to limit confounding variables. Specifically, males have a lower sensitivity to experimentally induced pain (including that induced by ischaemia, i.e., BFR) compared to females (Bartley and Fillingim 2013), which could subsequently influence RPE and the perceptual regulation of two experimental conditions. Additionally, changes in oestrogen across the menstrual cycle are associated with corresponding changes in endothelial function (Moreau et al. 2013), influencing arterial inflow (i.e., hypoxia) and venous outflow (i.e., metabolic accumulation). Because of these reasons, only one sex was recruited.

### 3.2 Preliminary Visit

The preliminary visit involved determining participants’ individualised BFR pressure, executing an incremental cycling test to exhaustion and familiarising participants with cycling with BFR. Upon arrival, participants rested supine for 10 min, after which measurements of brachial blood pressure (HEM‐7203, Omron, Australia) and the circumference of the right thigh (33% from the inguinal crease to the superior border of the patella) were recorded. These measurements were then incorporated into an established equation (Loenneke et al. 2015) to estimate arterial occlusion pressure. Arterial occlusion was estimated as the Hokanson system could not produce sufficient pressure to induce arterial occlusion with 5‐cm cuffs during piloting and wider cuffs negatively impacted cycling technique (Smith et al. 2022).

Participants then performed a 5‐min self‐selected warm‐up followed by an incremental cycling test to determine maximal oxygen uptake, peak aerobic power and the first and second ventilatory thresholds. The test involved 1‐min stages on a cycle ergometer (Velotron, RacerMate) beginning at 50 W with a 25‐W·min^−1^ increase until volitional exhaustion or when cadence dropped below 60 rpm for 5 s. Ventilatory gases were measured via a metabolic cart (TrueOne 2400, ParvoMedics; test‐retest coefficient of variation for V̇O_2_ = 4.7% (Crouter et al. 2006)). Maximal oxygen uptake was calculated as the average of the two highest consecutive 15‐s mean values. Two exercise physiologists independently determined the first and second ventilatory thresholds (VT_1_ and VT_2_): VT_1_ was identified as a sudden rise in V̇E/V̇O_2_ with no increase in V̇E/V̇CO_2_, whereas VT_2_ was determined by an exponential increase in both V̇E/V̇O_2_ and V̇E/V̇CO_2_ (Lucía et al. 2000). Discrepancies were resolved by consulting a third assessor. Peak aerobic power was calculated as the power of the last completed stage plus a pro‐rata value of the final stage (Peiffer et al. 2008). Finally, participants were familiarised with experimental procedures using 10 min of self‐paced cycling with BFR.

### 3.3 Experimental Sessions

Experimental sessions began with a 5‐min warm‐up at 50% of the power output associated with VT_1_ (maintained by the Velotron software), followed by 3 min of passive rest and a 10‐min experimental bout. During BFR_PWR_ and CON_PWR_ (i.e., fixed‐power trials), the Velotron software maintained the workload during the 10‐min bout at the power associated with VT_1_. Participants reported RPE, effort, muscular discomfort and cuff pain every 2 min. During BFR_RPE_ and CON_RPE_, the workload was freely adjustable (via changing virtual gears). Participants reported effort, muscular discomfort and cuff pain every 2 min and were instructed to cycle at the RPE reported at the end of that 2‐min period during CON_PWR_ (i.e., the RPE reported at minute 4 was used to prescribe workload during minutes 2–4). For BFR sessions only, 5‐cm‐wide pneumatic cuffs (5CS, Hokanson, USA) were applied to the proximal thighs after the warm‐up. The cuffs were instantaneously inflated (E20 inflator and AG101 air source, Hokanson, USA) to 60% of arterial occlusion pressure (173 ± 12 mmHg) immediately before the 10‐min bout. For all trials, participants were instructed to maintain the same constant self‐selected cadence as during CON_PWR_ and thus were blind to all measurements (e.g., power output and heart rate) except cadence and time elapsed. Fingertip blood lactate concentration was measured 15 s prior, immediately after and 2 min following the 10‐min bout using a handheld analyser (Lactate Pro II, Arkray). Power output was measured by a power meter (InfoCrank, Verve Cycling; coefficient of variation in precision = 0.6 ± 0.4% (Maier et al. 2017)) fitted to the Velotron. Heart rate (HRM‐Dual, Garmin) and power output were recorded continuously by a cycling computer (130 Edge, Garmin). Both V̇O_2_ and respiratory frequency were measured using the TrueOne 2400 metabolic cart, with data averaged into 2‐min mean values. A fan (1 meter in diameter) producing a wind speed of 32 km·h^−1^ was placed 2 meters in front of the bike.

Participants' RPE, perceived effort, muscular discomfort, and cuff pain were obtained using separate 11‐point numeric scales ranging from 0 (‘nothing at all’) to 10 (‘maximal’), except for effort, which ranged from 0% (‘nothing at all’) to 100% (‘maximal’). Borg's CR‐10 (G. A. V. Borg 1982) scale was used to obtain RPE, whereas all other scales were constructed using the same numbers and similar anchors (i.e., 3 = ‘*moderate*’). Each perceptual scale was explained during familiarisation, and the definition of each metric was restated at the beginning of each experimental session. Participants were instructed that RPE was ‘*a measure of whole‐body physical exertion and should encompass cardiovascular demands, or the sense of “breathlessness*,” *as well as sensations in the muscles of the legs caused by exercise and other sensations associated with exertion*’ (Peñailillo et al. 2018). Perceived effort was defined as ‘*the amount of mental or physical energy being given to complete the task. It is the overall effort needed to maintain the intensity of the exercise*’ (du Plessis et al. 2020). Briefly, RPE measures overall physical strain, whereas perceived effort quantifies the relative mental and physical resources required to maintain the task (Abbiss et al. 2015). Muscular discomfort was defined as ‘*any uncomfortable sensation within the leg muscles associated with exercise*’. Cuff pain was defined as ‘*the intensity of pain experienced specifically from the BFR cuffs compressing your thigh*. *This includes any type of pain*, *such as sharp*, *dull*, *or throbbing pain*’.

### 3.4 Statistical Analysis

Linear mixed models were used to examine differences for all variables, with participants included as a random factor. Models examining differences in power output during fixed‐RPE trials and RPE during fixed‐power trials included fixed effects of BFR (two levels: with and without) and time (five levels: 2, 4, 6, 8 and 10 min). The model for pain during BFR trials included fixed effects of time (five levels: 2, 4, 6, 8 and 10 min) and prescription method (two levels: fixed‐power and fixed‐RPE). Three‐factor models to examine differences in V̇O_2_, respiratory frequency, heart rate, blood lactate, effort and muscular discomfort included BFR (two levels: with and without), time (five levels: 2, 4, 6, 8 and 10 min) and prescription method (two levels: fixed‐power and fixed‐RPE). Main and interaction effects were examined using the Holm–Bonferroni method. Effect sizes were calculated as Cohen’s *d*_z_ using mean values of the session, timepoint or condition as appropriate. Pearson correlation coefficients with 95% confidence intervals (CI: lower limit, upper limit) were used to examine the association between the difference (BFR *minus* unrestricted) of all variables and RPE during fixed‐power trials or power output during fixed‐RPE trials. Analyses were performed using jamovi (v2.0.0) with significance set at ≤ 0.05. Data are presented as mean ± standard deviation, except in‐text when presented as mean percent change ± standard deviation of the percent change (BFR *minus* unrestricted).

## 4 Results

The results of the incremental cycling test to exhaustion were as follows: maximal oxygen uptake = 39.2 ± 8.2 mL·kg^−1^·min^−1^; maximal heart rate = 182 ± 17 beats·min^−1^ and the heart rate associated with VT_1_ = 146 ± 20 beats·min^−1^ and VT_2_ = 169 ± 20 beats·min^−1^; peak aerobic power output = 283 ± 49 W and the power output associated with VT_1_ = 161 ± 38 W and VT_2_ = 242 ± 54 W. Based on these results, the warm‐up for all sessions was completed at 81 ± 19 W. For fixed‐power trials (CON_PWR_ and BFR_PWR_), the 10‐min experimental bout was performed at 161 ± 38 W. Both perceptually regulated trials (CON_RPE_ and BFR_RPE_) were prescribed at an RPE of 2.3 ± 1.3 au (0–2 min), 3.2 ± 1.2 au (2–4 min), 3.4 ± 1.3 au (4–6 min), 3.5 ± 1.4 au (6–8 min) and 4.0 ± 1.6 au (8–10 min), based on the RPE reported during CON_PWR_.

### 4.1 Performance

As shown in Table 1, power output (a two‐way [BFR by time] analysis between BFR_RPE_ and CON_RPE_) was 22 ± 27% lower (*p* < 0.001, *d*_z_ = 0.9) during BFR_RPE_ compared to CON_RPE_ and increased over time (*p* < 0.001) without an interaction (*p* = 0.865).

**TABLE 1:** Power output and perceptual responses during 10 min of cycling at a fixed‐power (PWR) and fixed‐rating of perceived exertion (RPE) both with blood flow restriction (BFR) and without (CON).

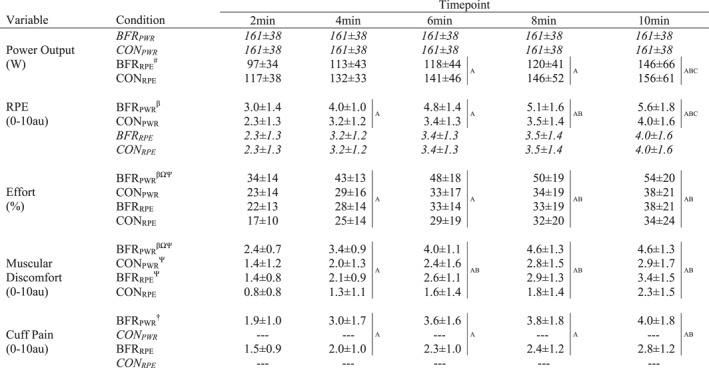

*Note:* BFR_PWR_ = fixed‐power with BFR, CON_PWR_ = fixed‐power without BFR, BFR_RPE_ = fixed‐RPE with BFR, CON_RPE_ = fixed‐RPE without BFR. Fixed‐power was set at the power output associated with the first ventilatory threshold. Fixed‐RPE trials were prescribed at the same RPE reported at each 2‐min period during CON_PWR_. Data presented as mean ± standard deviation. Data in *italics* were not included in the analysis as they were used to prescribe workload for that metric. — indicates no data available due to the condition. Main effect of time: ^A^ greater than 2 min, ^B^ greater than 4 min, ^C^ greater than 6 min. Main effect of condition: ^†^ BFRpower > BRFrpe, ^#^ CONrpe > BFRrpe. Condition × prescription interactions: ^β^ greater than CON_PWR_, ^Ω^ greater than BFR_RPE_, ^Ψ^ greater than CON_RPE_.

### 4.2 Physiological Responses

**FIGURE 1:**
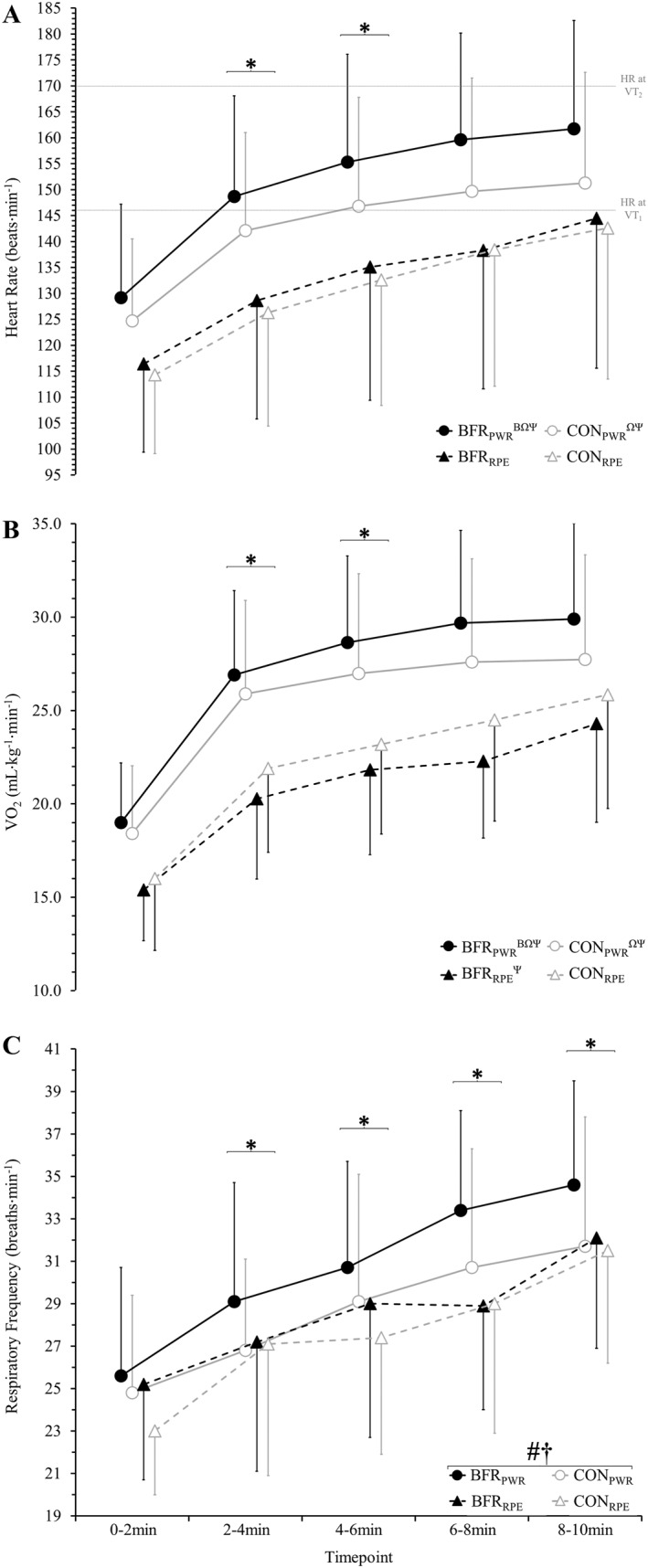
Mean 2‐min heart rate (A), oxygen consumption (V̇O_2_; B) and respiratory frequency (C) during 10 min of cycling. Four conditions were completed, involving 10 min of cycling: with blood flow restriction (BFR) and without (CON) at a fixed power (BFR_PWR_ and CON_PWR_) and rating of perceived exertion (RPE; BFR_RPE_ and CON_RPE_) matched to CON_PWR_. * different to the previous timepoint (main effect of time). # BFR different to CON (main effect of BFR). † fixed‐power different to fixed‐RPE (main effect of prescription method). Interaction effect (BFR by prescription method): β different to CON_PWR_; Ω different to BFR_RPE_; Ψ different to CON_RPE_.

Heart rate displayed a two‐way interaction (*p* = 0.010; Figure 1A) between BFR application (i.e., with BFR vs. without) and prescription method (i.e., fixed‐power vs. fixed‐RPE). Heart rate was greater during BFR_PWR_ (151 ± 23 beats·min^−1^) compared to all other conditions (CON_PWR_: 143 ± 22 beats·min^−1^, *p* < 0.001, *d*_z_ = 0.7; BFR_RPE_: 133 ± 26 beats·min^−1^, *p* < 0.001, *d*_z_ = 1.2; CON_RPE_: 131 ± 25 beats·min^−1^, *p* < 0.001, *d*_z_ = 1.6). Additionally, CON_PWR_ was 9 ± 10% higher than BFR_RPE_ (*p* < 0.001, *d*_z_ = 0.8) and 10 ± 7% greater than CON_RPE_ (*p* < 0.001, *d*_z_ = 1.6) with no difference between BFR_RPE_ and CON_RPE_ (*p* = 0.298). A main effect of time was also noted, with heart rate increasing across all trials (*p* < 0.001). A three‐way interaction was not observed (*p* = 0.858).

The V̇O_2_ demonstrated a two‐way interaction between BFR application and prescription method (*p* < 0.001; Figure 1B). Greater V̇O_2_ was observed during BFR_PWR_ (26.8 ± 6.0 mL·kg^−1^·min^−1^) compared to all other conditions (CON_PWR_: 25.3 ± 6.0 mL·kg^−1^·min^−1^, *p* = 0.007, *d*_z_ = 1.2; CON_RPE_: 22.3 ± 5.9 mL·kg^−1^·min^−1^, *p* < 0.001, *d*_z_ = 1.2; BFR_RPE_: 20.8 ± 5.1 mL·kg^−1^·min^−1^, *p* < 0.001, *d*_z_ = 1.3). Additionally, CON_PWR_ was 15 ± 19% and 24 ± 28% greater compared to CON_RPE_ (*p* < 0.001, *d*_z_ = 0.9) and BFR_RPE_ (*p* < 0.001, *d*_z_ = 1.0), respectively, with CON_RPE_ 7 ± 10% greater compared to BFR_RPE_ (*p* = 0.007, *d*_z_ = 0.7). A main effect of time was also noted, with V̇O_2_ increasing across all trials (*p* < 0.001). A three‐way interaction was not observed (*p* = 0.710).

Respiratory frequency (Figure 1C) showed main effects of BFR (*p* < 0.001), prescription method (*p* < 0.001) and time (*p* < 0.001). When using BFR, respiratory frequency was greater (29 ± 6 vs. 28 ± 6 breaths·min^−1^, *p* < 0.001, *d*_z_ = 0.8) than without BFR, and respiratory frequency was greater during fixed‐power trials compared to fixed‐RPE (29 ± 6 vs. 28 ± 6 breaths·min^−1^, *p* < 0.001, *d*_z_ = 0.6). In all conditions, respiratory frequency increased continuously across trials (*p* ≤ 0.018, *d*_z_ = 1.1–2.3). No two‐way (BFR by timepoint: *p* = 0.990; BFR by prescription method: *p* = 0.116; timepoint by prescription method: *p* = 0.310) or three‐way interaction (*p* = 0.339) was observed.

**FIGURE 2:**
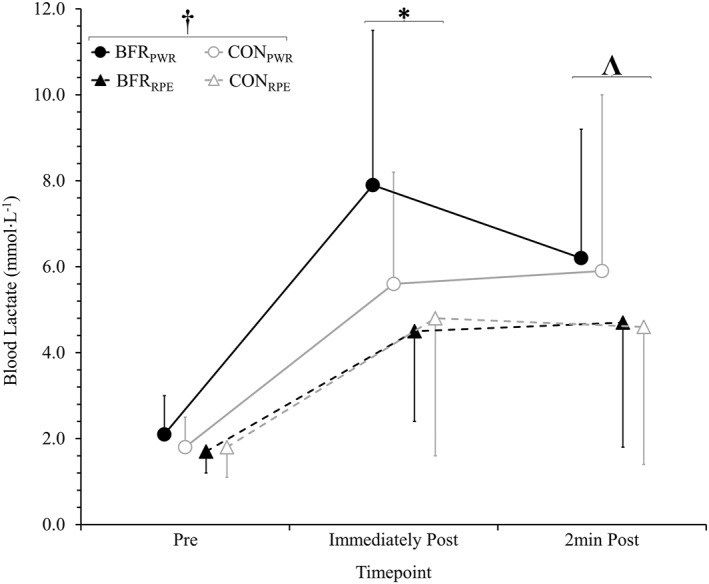
Blood lactate concentration 15 s prior (Pre), immediately after (Post) and 2 min after (2 min Post) 10 min of cycling. Four conditions were completed, involving 10 min of cycling: with blood flow restriction (BFR) and without (CON) at a fixed power (BFR_PWR_ and CON_PWR_) and rating of perceived exertion (RPE; BFR_RPE_ and CON_RPE_) matched to CON_PWR_. † fixed‐power different to fixed‐RPE (main effect of prescription method). * different to the previous timepoint. Λ different to pre.

Blood lactate displayed main effects of prescription method (*p* < 0.001) and timepoint (*p* < 0.001; Figure 2). Blood lactate was greater during fixed‐power (4.9 ± 3.5 mmol·L^−1^) compared to fixed‐RPE trials (3.6 ± 2.7 mmol·L^−1^, *p* < 0.001, *d*_z_ = 1.0). Pre‐exercise blood lactate (1.8 ± 0.7 mmol·L^−1^) was lower compared to immediately post‐exercise (5.7 ± 3.1 mmol·L^−1^, *p* < 0.001, *d*_z_ = 1.6) and 2 min post‐exercise (5.4 ± 3.3 mmol·L^−1^, *p* < 0.001, *d*_z_ = 1.4). No difference was observed immediately post compared with 2 min post‐exercise (*p* = 0.411). No main effect of BFR (*p* = 0.192), two‐way interactions (BFR by timepoint: *p* = 0.445; BFR by prescription method: *p* = 0.091; timepoint by prescription method: *p* = 0.066) or three‐way interactions (*p* = 0.217) were observed.

### 4.3 Perceptual Responses

Perceptual responses are shown in Table 1. The RPE (a two‐way [prescription method by time] analysis between BFR_PWR_ and CON_PWR_) was 0.7 ± 0.4 au greater (*p* < 0.001, *d*_z_ = 1.5) with BFR_PWR_ compared to CON_PWR_ and increased over time (*p* < 0.001) without an interaction (*p* = 0.075). Perceived effort increased over time for all conditions (*p* < 0.001) with an interaction between BFR by prescription method (*p* < 0.001): BFR_PWR_ was greater compared to all other conditions (CON_PWR_: *p* < 0.001, *d*_z_ = 1.5; BFR_RPE_: *p* < 0.001, *d*_z_ = 1.6; CON_RPE_: *p* < 0.001, *d*_z_ = 1.9). Muscular discomfort increased over time in all conditions (*p* < 0.001) with an interaction between BFR by prescription method (*p* < 0.001): BFR_PWR_ was greater compared to all other conditions (CON_PWR_: *p* < 0.001, *d*_z_ = 1.1; BFR_RPE_: *p* < 0.001, *d*_z_ = 1.8; CON_RPE_: *p* < 0.001, *d*_z_ = 1.6); additionally, muscular discomfort was lower during CON_RPE_ compared to CON_PWR_ (*p* < 0.001, *d*_z_ = 1.2) and BFR_RPE_ (*p* < 0.001, *d*_z_ = 1.1); there was no difference between CON_PWR_ and BFR_RPE_ (*p* = 0.283). Cuff pain was 1.1 ± 1.2 au greater (*d*_z_ = 0.9) during BFR_PWR_ compared to BFR_RPE_ (*p* < 0.001) and increased throughout both trials (*p* < 0.001) without an interaction (*p* = 0.106).

**FIGURE 3:**
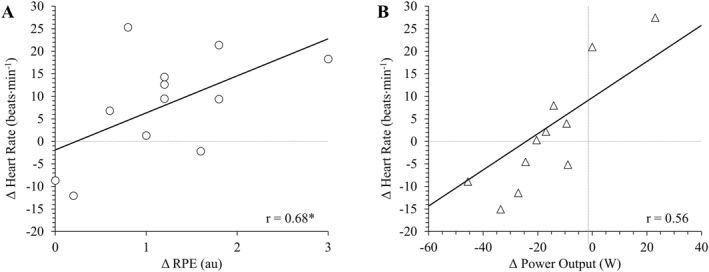
Correlations between the difference in average heart rate versus (A) difference in average rating of perceived exertion (RPE) during fixed‐power trials and (B) difference in average power output during fixed RPE trials. Difference scores are calculated by subtracting the trial with blood flow restriction from the trial without restriction. * significant correlation (*p* = 0.015).

For fixed‐power trials, the difference (BFR *minus* CON) in RPE was correlated with heart rate (*r* = 0.68; CI = 0.17, 0.90; *p* = 0.015; Figure 3A). The difference in RPE was not correlated with V̇O_2_ (*r* = 0.54; CI = −0.05, 0.85; *p* = 0.068), respiratory frequency (*r* = 0.32; CI = −0.31, 0.76; *p* = 0.305), blood lactate either immediately (*r* = 0.49; CI = −0.12, 0.83; *p* = 0.107) or 2 min post‐exercise (*r* = 0.52; CI = −0.08, 0.84; *p* = 0.085), or muscular discomfort (*r* = 0.26; CI = −0.37, 0.72; *p* = 0.424).

For fixed‐RPE trials, the difference (BFR *minus* CON) in power output was not correlated with any variable (V̇O_2_: *r* = 0.02; CI = −0.56, 0.59; *p* = 0.941; respiratory frequency: *r* = 0.31; CI = −0.32, 0.75; *p* = 0.327; heart rate: *r* = 0.56; CI = −0.02, 0.86; *p* = 0.058; blood lactate immediately post‐exercise: *r* = 0.09; CI = −0.51, 0.63; *p* = 0.078; blood lactate 2 min post‐exercise: *r* = 0.24; CI = −0.39, 0.72; *p* = 0.450; muscular discomfort: *r* = 0.38; CI = −0.25, 0.78; *p* = 0.221).

## 5 Discussion

This study examined recreationally active males' performance, physiological and perceptual responses to 10 min of cycling with and without BFR at both fixed‐power and fixed‐RPE. Consistent with the hypothesis, applying BFR during fixed‐power cycling at VT_1_ resulted in greater physiological and perceptual responses compared to a perceptually regulated approach. Additionally, BFR_RPE_ elicited lower physiological demands without altering perceptual responses compared to CON_PWR_. As such, the chosen prescription method influences the physiological and perceptual responses during BFR cycling, which has implications for selecting a fixed‐power or fixed‐RPE approach.

The BFR_PWR_ condition resulted in the greatest physiological demands of all conditions examined, which has implications for exercise prescription. The magnitude of the cardiovascular stress during BFR_PWR_ has previously been shown to improve aerobic fitness in recreationally active individuals cycling with BFR for 15 min three times per week (Abe et al. 2010). As such, a fixed‐power approach is likely to be used as a time‐effective method of prescribing BFR exercise to improve cardiovascular health. However, the elevation in heart rate during BFR_PWR_ was greater than anticipated, and thus the associated cardiac stress requires consideration, particularly in clinical populations due to the elevated blood pressure (i.e., pressor response) (Spranger et al. 2015). It was observed that heart rate during the last 2 min of BFR_PWR_ exceeded the heart rate associated with VT_2_ in one third of participants (relative difference: +10 to 21 beats·min^−1^). The exercise intensity for these participants therefore shifted from the *moderate* to the *heavy* domain (i.e., from between VT_1_ and VT_2_ to > VT_2_) (Burnley and Jones 2007). For comparison, only one participant's heart rate exceeded the heart rate associated with VT_2_ (+7 beats·min^−1^) during CON_PWR_. These observations support previous work (Ozaki et al. 2010; Kilgas et al. 2022) that BFR exercise can exacerbate physiological measures of intensity for a given external intensity. This demonstrates that a fixed‐power approach may not accurately prescribe the physiological stimulus of aerobic BFR exercise in healthy adults. It is also worth examining if caution is needed when prescribing fixed‐power BFR to populations for whom high cardiac stress (i.e., heavy exercise intensities) is not recommended, such as individuals with cardiovascular disease or chronic obstructive pulmonary disease. Nevertheless, if high physiological stress is desired at moderate mechanical loads, BFR_PWR_ is a suitable alternative for CON_PWR_, which could be specifically investigated in individuals with load‐compromised joints and bones, such as those with arthritis or osteoporosis (Ferguson 2014). An additional consideration for BFR_PWR_ is the corresponding greater muscular discomfort and cuff pain compared to CON_PWR_, which could reduce exercise adherence (Ekkekakis et al. 2011). As such, the physiological demands of fixed‐power BFR cycling should be weighed against the heightened discomfort and pain, particularly in populations where such perceptual responses are undesirable.

Both V̇O_2_ and heart rate during BFR_RPE_ and CON_RPE_ were lower compared to CON_PWR_, despite all three conditions being performed at the same RPE. As such, in recreationally active males, a fixed‐RPE prescription method does not elicit the same cardiovascular demands as the fixed‐power approach used to derive the RPE. This difference in cardiovascular demands at the same RPE contradicts previous literature in athletic populations (Smith et al. 2024). Specifically, no difference in heart rate or blood lactate has been shown cycling at the power associated with VT_1_ (i.e., CON_PWR_) compared to a fixed‐RPE with BFR condition (i.e., BFR_RPE_) in trained cyclists (Smith et al. 2024). The difference between these populations is likely due to the current participants being recreationally active and not trained cyclists with extensive self‐paced cycling experience (St Clair Gibson et al. 2006; Tucker 2009). A given RPE is associated with a particular magnitude and type of physiological and perceptual strain (St Clair Gibson et al. 2006), which is likely different when an exercise modality is novel (e.g., running vs. cycling). For instance, greater experience with running has been shown to improve the ability to reproduce a particular RPE, especially at low to moderate exercise intensities (Borg 6–20 = 9 and 13, respectively) (Eston and Williams 1988), similar to those prescribed in this study. As such, an RPE‐based prescription model will likely result in lower physiological stress compared to the same RPE reported during a fixed‐power approach for healthy adults exercising at a moderate exercise intensity.

The inability to match physiological demands appears to be irrespective of BFR application. This is indicated by the lack of correlations between the difference in power output during fixed‐RPE trials and any measured variable (Figure 3B). This contradicts literature showing that the power output of trained cyclists during perceptually regulated BFR cycling is closely associated with muscular discomfort induced by BFR (Smith et al. 2024). This discrepancy with previous literature indicates that the BFR‐induced responses on RPE could be population‐dependent, warranting further research to use BFR as a tool to improve our understanding of how external stressors influence RPE in various cohorts (St Clair Gibson et al. 2006). Nevertheless, BFR‐related sensations likely have no greater influence on RPE compared to non‐BFR exercise‐related sensations in recreationally active males. Indeed, heart rate, respiratory frequency and blood lactate were not different between BFR_RPE_ and CON_RPE_ (Figures 1 and 2), indicating that BFR did not compromise physiological stress during fixed‐RPE cycling. The practical implication is that although BFR heightens RPE at fixed workloads, BFR‐induced stress does not impede the use of a perceptually regulated approach in recreationally active individuals. Overall, these findings indicate that BFR is not a concern when using an RPE‐based prescription method with healthy adults.

Cuff pain was greater during BFR_PWR_ compared to BFR_RPE_ despite the same exercise duration and cuff pressure being used; the only difference was exercise intensity and thus RPE. Whole‐body RPE integrates central (i.e., heart rate and ventilation) (Nicolò et al. 2014; Ekblom and Golobarg 1971) and peripheral (e.g., metabolic acidosis) (G. Borg et al. 1987) feedback, with perceptual stress including exercise‐related sensations such as discomfort and pain (St Clair Gibson et al. 2006). Each of these responses is increased during aerobic BFR exercise at a given workload (Thomas et al. 2018; Corvino et al. 2017), indicating that all aspects of BFR‐induced stress are represented by RPE. Additionally, no single construct is the primary determinant of RPE (Abbiss and Laursen 2005), indicating that the greatest stressor at any given time will contribute the most to momentary RPE. This is advantageous for prescribing BFR exercise to healthy adults, as the magnitude of each stressor varies based on the combination of cuff pressure and duration as well as exercise intensity and structure (i.e., continuous or interval) (Smith et al. 2021). The use of RPE to prescribe BFR exercise is therefore advantageous as it reliably represents the overall physiological and perceptual stress, including cuff pain, in healthy and clinical populations (Chen et al. 2002). Thus, an RPE prescription model is a convenient method of setting exercise intensity to balance the physiological and perceptual demands associated with BFR.

This study is not without limitations. First, the small sample size, while providing adequate power to detect differences (*ƒ* = 0.35), does increase the likelihood of random errors such as those due to individual differences. No outliers were identified, and thus the findings are likely to represent the population, yet additional investigations should be used to verify the conclusions. Second, CON_PWR_ was always performed first to allow meaningful comparisons, yet this could introduce the potential for a priming effect. Future research could explore the order effects of exercise with versus without BFR to further validate the present findings. Third, fixed‐RPE conditions were only matched to CON_PWR_ and not BFR_PWR_, thus preventing a complete analysis of the physiological and perceptual differences between fixed‐RPE and fixed‐power prescription methods. However, the inclusion of two additional conditions was not statistically or practically feasible and would compromise the focus of this research. Fourth, only one sex was recruited to prevent confounding variables influencing the study outcomes. Briefly, males have lower sensitivity to experimentally induced pain compared to females (Bartley and Fillingim 2013), and changes in oestrogen across the menstrual cycle affect endothelial function (Bartley and Fillingim 2013), which could in turn impact the BFR stimulus and RPE‐based regulation of exercise intensity. Although including only one sex limits the generalisability of findings, it maintained the robustness of the conclusions. Fifth, recent studies have reported the estimation of arterial occlusion pressure is not accurate in some individuals when using 5‐cm‐wide cuffs on the legs (Yamada et al. 2023; Spitz et al. 2024). An appropriate alternative solution does not yet exist for circumstances where wider cuffs are unsuitable; this warrants further research. Finally, the acute nature of this study prevents conclusions on the long‐term use of fixed‐power and perceptually regulated cycling to achieve a given outcome; thus, relevant training interventions are warranted.

## 6 Conclusion

Aerobic BFR exercise is an effective training tool for healthy adults, yet the decision to use BFR and selection of a prescription approach (fixed‐power vs. fixed‐RPE) depends on the desired outcome. The decision to use BFR in any population depends on the likelihood of adherence, as this technique induces greater discomfort and cuff pain compared to performing the same task without BFR. For instance, although BFR_PWR_ elicits the greatest physiological responses, the associated discomfort and pain could be a barrier to exercise; thus, the cuff pain associated with BFR is important for adherence. To reduce discomfort and pain, one might consider decreasing the power output (or cuff pressure), although this adjustment will inevitably reduce the physiological stimulus as well. If choosing to use BFR, the difficulty of accurately prescribing physiological intensity within a fixed‐power model (even if using individualised intensity thresholds and cuff pressures) makes this method potentially unsafe for individuals when high cardiac stress is contraindicated. A potentially safer alternative is a fixed‐RPE approach, as RPE reflects the overall physiological and perceptual stress. Indeed, the fixed‐RPE prescription method caused less discomfort and pain than BFR_PWR_ without compromising physiological stress compared to CON_RPE_. A fixed‐RPE approach is also convenient, as the RPE from a non‐BFR session can be used to prescribe exercise of similar duration with BFR. Therefore, a fixed‐RPE approach may also present a suitable initial method for introducing aerobic BFR exercise before progressing to fixed‐power cycling. Ultimately, when prescribing aerobic BFR exercise, it becomes crucial to consider these issues surrounding both fixed‐power and fixed‐RPE prescription models in relation to the desired outcomes.

## Conflicts of Interest

The authors declare no conflicts of interest.
